# Compressive Deformation, Damage Evolution, and Energy Absorption of 3D-Printed PLA and Short-Carbon-Fiber-Reinforced PLA Auxetic Metamaterials

**DOI:** 10.3390/polym18172173

**Published:** 2026-09-06

**Authors:** Lan Luo, Shidian Qiu, Maokai Li, Lianchao Wang, Zhengxian Liu

**Affiliations:** 1 National Key Laboratory of Science and Technology on Advanced Composites in Special Environments, Harbin Institute of Technology (HIT), Harbin 150080, China; 2 Guangzhou Future Additive Manufacturing Research Institute, Guangzhou 510150, China; 3 Department of Materials Science, Polytechnic University of Madrid, 28040 Madrid, Spain

**Keywords:** short-carbon-fiber composite, fused deposition modeling, polylactic acid, auxetic metamaterial, energy absorption

## Abstract

Auxetic mechanical metamaterials convert axial compression into lateral contraction, providing deformation modes that are attractive for lightweight energy absorbers. Here, fused deposition modeling was used to fabricate polylactic acid (PLA) and 10 wt% short-carbon-fiber-reinforced PLA (CF/PLA) auxetic metamaterials with arrow, re-entrant hexagonal, star-shaped, and chiral rotating topologies. Thermal analysis, tensile testing, and three-point bending first established the effect of carbon-fiber addition on the printable matrix. Quasi-static compression experiments were then combined with finite-element simulations using pressure-dependent plasticity and ductile damage to resolve topology-dependent collapse and energy partition. Adding 10 wt% short carbon fibers increased the tensile modulus from 2.33 to 3.72 GPa and raised the plateau stresses of the auxetic structures by about 50%. The star-shaped CF/PLA metamaterial showed the highest specific energy absorption (approximately 4.7 J/g), whereas the chiral rotating topology showed the largest relative improvement, exceeding 130%. Fiber reinforcement improved stiffness and load transfer but promoted stress localization at hinges, re-entrant corners, and ligament junctions. These findings elucidate the material–topology trade-off between stiffness enhancement and localized embrittlement, offering practical guidelines for designing crashworthy 3D-printed composite metamaterials.

## 1. Introduction

Auxetic mechanical metamaterials are architected solids whose effective Poisson’s ratio is governed by cell geometry rather than by the intrinsic Poisson’s ratio of the base material [1,2,3]. When stretched, they expand in the transverse direction, whereas compression causes an inward contraction toward the loading axis. This behavior is commonly associated with the bending of re-entrant ribs, rotation of ligaments, hinge-like motion at nodes, and local instability of the cell walls. Owing to these deformation modes, auxetic lattices can exhibit improved indentation resistance, shear rigidity, fracture tolerance, and energy absorption compared with conventional cellular solids [4,5,6]. Their response can be tuned by varying the re-entrant angle, wall thickness, relative density, and cell gradient. These parameters govern the stiffness, peak load, plateau stress, and densification strain [7,8,9,10]. Such characteristics are attractive for aerospace sandwich panels, crashworthy unmanned-aircraft components, satellite protection structures, landing attenuators, and other lightweight systems in which high energy absorption is required with minimal added mass [11,12,13,14,15,16].

Additive manufacturing has made these complex cellular geometries easier to realize. Among the available processes, fused deposition modeling (FDM) is widely used for thermoplastic auxetic structures because it is inexpensive, flexible in design, and suitable for rapid fabrication of customized lattices [17,18]. Polylactic acid (PLA) is a common feedstock for this purpose due to its low processing temperature, small warpage, and reliable dimensional accuracy [19,20,21]. Nevertheless, its relatively low toughness, limited thermal resistance, pronounced interlayer anisotropy, and tendency for rapid crack growth restrict its use in highly loaded energy-absorbing components [22,23]. Short carbon fibers are often introduced to improve stiffness, flexural resistance, and dimensional stability by promoting load transfer and constraining polymer-chain mobility [24]. This modification is not without drawbacks, since fiber agglomeration, void formation, weak interlayer bonding, and local stress concentration may occur. Consequently, the key issue is not simply whether CF/PLA is mechanically stronger than pure PLA, but how the reinforcement modifies the topology-dependent deformation and failure mechanisms responsible for the auxetic response [25,26,27].

Recent studies on additively manufactured PLA and PLA-composite auxetics have moved from single-cell geometries toward coupled material-architecture design. Kaya et al. [28] compared several 3D re-entrant cells and reported improved energy absorption in cross-ribbed structures. Bozorgnia et al. [29] showed that iron-powder/PLA re-entrant structures can substantially increase specific energy absorption relative to unfilled PLA. Shahmorad et al. [30,31] investigated hybrid PLA auxetic metamaterials under compression and impact and found that combined honeycomb, chiral, and cubic motifs can provide large negative Poisson’s ratios and high specific energy absorption. For CF/PLA components, Daniel et al. [32] showed that chiral and rotating-cell layouts can improve the mechanical performance of bone-plate-like structures. These studies establish the promise of topology modification and matrix reinforcement. Although previous studies have investigated individual auxetic topologies and fiber-reinforced structures, systematic comparisons across different auxetic metamaterials remain limited. In this study, four representative auxetic topologies were compared under consistent fabrication and loading conditions. Experimental characterization and finite-element analysis were combined to evaluate the effects of short-carbon-fiber reinforcement on deformation, damage, and energy-absorption behavior.

In this study, pure PLA and 10 wt% CF/PLA filaments were prepared by melt blending and extrusion and printed into four auxetic metamaterial topologies: arrow, re-entrant hexagonal, star-shaped, and chiral rotating cells. The base materials were characterized by thermogravimetric analysis, differential scanning calorimetry, uniaxial tension, and three-point bending. The printed metamaterials were compressed quasi-statically under identical loading conditions. The measured collapse modes were compared with ABAQUS simulations incorporating pressure-dependent plasticity, ductile-damage initiation, stiffness degradation, and element deletion. Peak stress, plateau stress, absorbed energy, and specific energy absorption were then used to compare the structures.

## 2. Material Fabrication

**Figure 1:**
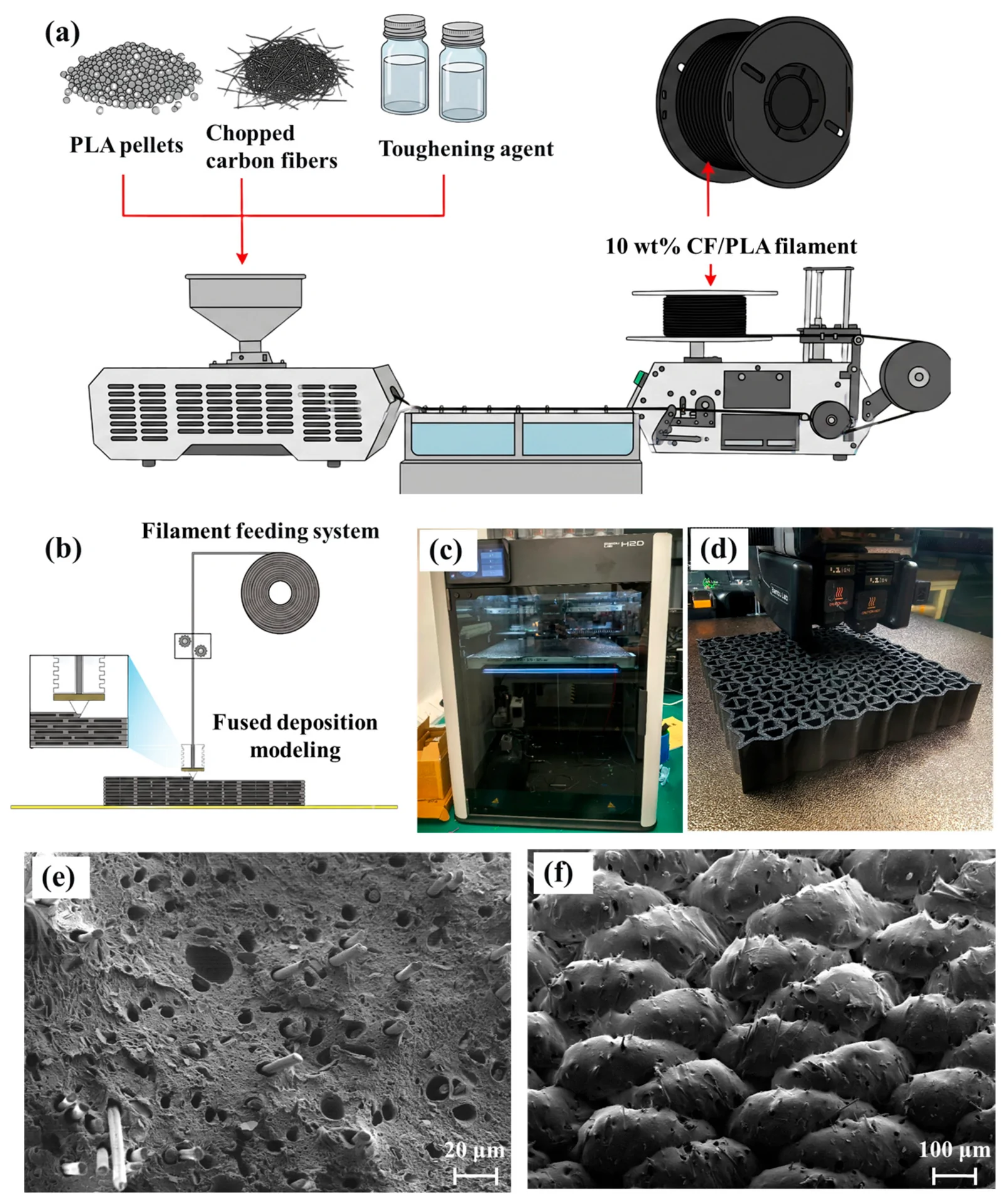
Printing preparation route and printing morphology observation: (**a**) carbon-fiber-reinforced filament preparation; (**b**) printing principle; (**c**,**d**) printer and printing process; (**e**) cross-sectional morphology of 10 wt% CF/PLA filament; (**f**) cross-sectional morphology of 10 wt% CF/PLA printed specimen.

PLA, short carbon fibers, and a toughening agent were used as the feedstock materials. PLA pellets (REVODE190) were supplied by Zhejiang Hisun Biomaterials Co., Ltd. (Taizhou, China). According to the manufacturer’s technical data sheet, the PLA had a density of 1.25 ± 0.05 g/cm^3^, a melting temperature of 170–180 °C, a glass-transition temperature of 60–63 °C, a tensile strength of 50 MPa. Chopped carbon fibers with a supplier-specified nominal cut length of 3.0 ± 0.5 mm were supplied by Dezhou Junteng Material Technology Co., Ltd. (Dezhou, China). The supplier-reported carbon-fiber properties were a carbon content of 95%, tensile strength of 3.5 GPa, tensile modulus of 220 GPa, density of 1.75 g/cm^3^, filament diameter of 7–10 μm. An acrylic core-shell copolymer elastomer impact modifier (Kane Ace B-564, Kaneka Corporation (Osaka, Japan); density: 1.10 g/cm^3^, particle size: 150–200 nm) was used as the toughening agent. It was incorporated at a fixed concentration of 5 wt% solely into the short-carbon-fiber-reinforced PLA formulation to promote shear yielding and improve fiber–matrix interfacial bonding. The printing preparation route and printing morphology observation are shown in Figure 1. Before compounding, raw materials were vacuum-dried at 55 °C for 4 h. The mixture was melt-compounded at 205 °C with a screw speed of 120 rpm (residence time: ~2 min). The dried pellets were extruded at 210 °C (15 rpm) at an extrusion rate of 18 g/min. The extruded filaments were cooled in a 25 °C water bath to obtain a diameter of 1.75 ± 0.03 mm.

The auxetic geometries were modeled in three dimensions and imported into slicing software to generate the FDM toolpaths. Specimens were fabricated using an H2DC-series FDM printer from Shenzhen Bambu Lab Technology Co., Ltd. (Shenzhen, China). The specimens were fabricated with a nozzle temperature of 230 °C, a build-plate temperature of 55 °C, a layer thickness of 0.30 mm, a printing speed of 200 mm/s, and a 100% solid infill. A single 0° raster direction was used during layer deposition to reduce the influence of toolpath orientation on the mechanical and energy-absorption comparisons. The specimens were cooled to room temperature after printing. Excess material was removed with scissors, and the remaining contours and burrs were lightly sanded before mechanical testing. Microstructures were examined using scanning electron microscopy (SEM) after gold sputtering. Representative SEM images of the 10 wt% CF/PLA filament and printed specimen are shown in Figure 1e,f. Local pores, inter-bead gaps, and fiber heterogeneity are visible, suggesting preferential fiber orientation along the extrusion and deposition directions.

## 3. Auxetic Metamaterial Design

**Figure 2:**
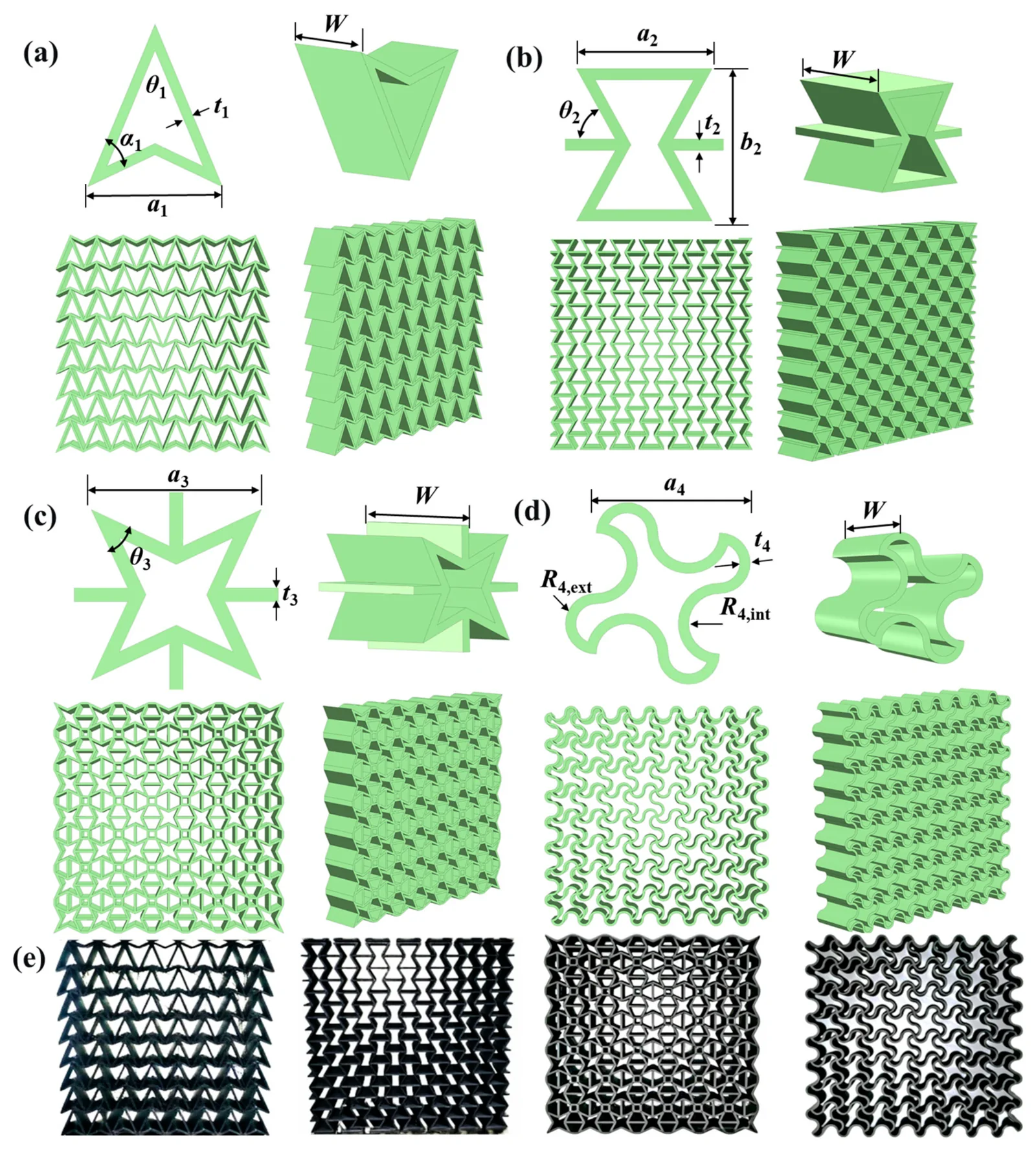
Four auxetic metamaterial designs: (**a**) arrow topology; (**b**) re-entrant hexagonal topology; (**c**) star-shaped topology; (**d**) chiral rotating topology; (**e**) printed specimens.

Four periodic auxetic topologies were designed to compare the influence of deformation mechanism on the load-bearing and energy-absorption behavior of PLA and CF/PLA metamaterials (Figure 2). The arrow topology consists of nested triangular inclined struts whose tips form flexible rotation zones. Compression is therefore accommodated by coupled strut rotation and progressive folding. The re-entrant hexagonal topology is a canonical auxetic cell in which inclined ribs rotate inward under compression, producing a negative Poisson’s ratio mechanism and directional load transfer. The star-shaped topology contains radially arranged re-entrant ligaments that contract laterally through combined arm rotation and bending while redistributing load in multiple directions. The chiral rotating topology connects neighboring nodes through continuous curved ligaments, so cell rotation and ligament bending generate a relatively smooth deformation path.

The four auxetic metamaterials were parametrically constructed by first defining the geometric dimensions, cell angles, and wall thickness (1.0 mm) of individual unit cells, which were then periodically tessellated into integer arrays to form specimens with nominal bounding dimensions close to 100 mm × 100 mm × 20 mm (Table 1). Because the different topologies resulted in different specimen masses, identical mass was not imposed among the four structures. Therefore, specific energy absorption (SEA) was adopted as the primary mass-normalized metric for cross-topology comparison.

## 4. Finite-Element Model for Quasi-Static Compression

**Figure 3:**
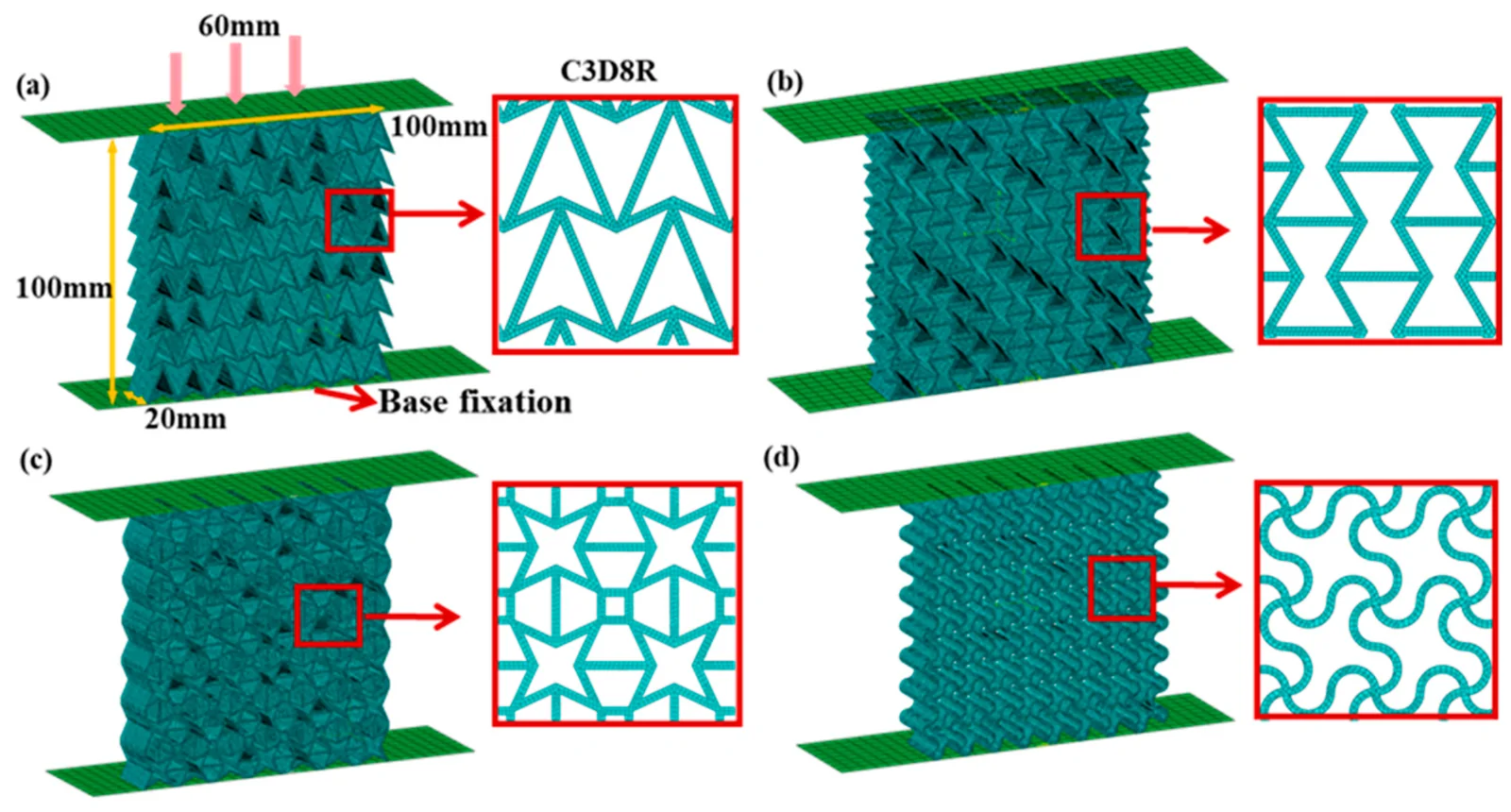
Finite-element compression models of the four metamaterials: (**a**) arrow; (**b**) re-entrant hexagonal; (**c**) star-shaped; (**d**) chiral rotating.

Quasi-static compression of the four metamaterial architectures was simulated in ABAQUS/Explicit 2024. The specimen thickness was 20 mm, and the initial separation between the rigid platens was 100 mm. The upper and lower platens were modeled using R3D4 discrete rigid elements and controlled through reference points. The lower platen was fully fixed, whereas the upper platen was constrained to move only in the compression direction. A displacement of 60 mm was applied to the upper platen over a total loading time of 60 s using a smooth-step amplitude to minimize inertial oscillations. The corresponding finite-element models of the four auxetic architectures are shown in Figure 3.

General contact was assigned between the platens and specimens and among the cell walls. The normal contact response used hard contact with separation allowed, and tangential contact was represented by a penalty friction coefficient of 0.2. Large deformation and self-contact were included. The linear Drucker–Prager and damage parameters were determined using reference mechanical data and calibrated against experimental responses. For pure PLA, reference compressive and tensile yield strengths of 69.49 MPa and 42.89 MPa were adopted [33], yielding a friction angle of *β* = 30° at a flow-stress ratio of *K* = 0.9. For 10 wt% CF/PLA, *K* = 1 was adopted, and *β* was calibrated to 20° against experimental compression curves. Dilation angles were set to 5° (PLA) and 10° (CF/PLA), while fracture energies were calibrated from post-peak softening as 300 J/m^2^ and 270 J/m^2^, respectively. Ductile-damage initiation and fracture-energy-based linear damage evolution were introduced to represent stiffness degradation and crack propagation during cell-wall crushing, with failed-element deletion enabled. Tensile-side damage parameters were converted from the measured tensile curves. To verify the quasi-static condition, the kinetic energy (ALLKE), internal energy (ALLIE), and artificial energy (ALLAE) were monitored. Throughout the compression, the ALLKE/ALLIE ratio remained below 1.92% (well within the widely accepted 5% threshold) [34], while the ALLAE/ALLIE ratio remained below 3%, confirming that inertial effects and artificial strain energy were negligible. Numerical stability and quasi-static validity were verified through enhanced hourglass control, mesh-convergence analyses, energy-balance tracking, and ABAQUS/Explicit second-order accuracy section controls. This setting enhances time-integration precision while maintaining the standard first-order interpolation of the C3D8R elements.

A mesh-convergence study was conducted using the chiral rotating topology with element sizes of 0.75 mm, 0.50 mm, and 0.25 mm (corresponding to 205,794, 505,800, and 3,922,960 elements, respectively). Using the 0.25 mm mesh as the reference and a 5% relative error criterion, the 0.50 mm mesh exhibited differences of only 1.00–4.26% across the monitored stress, strain, and energy outputs throughout compression. Balancing accuracy and computational cost, an element size of 0.50 mm was selected for all subsequent simulations, and the final mesh statistics are summarized in Table 2. The material parameters used in the finite-element simulations are summarized in Table 3.

## 5. Experimental

### 5.1. Thermal Characterization

Thermogravimetric analysis (TGA) and differential scanning calorimetry (DSC) were used to characterize the thermal stability and thermal transitions of pure PLA and 10 wt% CF/PLA. Before testing, the samples were dried in a vacuum oven to remove absorbed moisture. TGA was performed under nitrogen using approximately 5–10 mg of material in an alumina crucible. The nitrogen flow rate was 50 mL/min, and the samples were heated from 25 to 500 °C at 10 °C/min. The onset of decomposition, temperature of maximum mass-loss rate, and residual mass at 500 °C were obtained from the TGA and derivative thermogravimetric curves. DSC tests were also performed under nitrogen using 5–10 mg samples sealed in aluminum pans, with an empty pan as the reference. Samples were heated from 25 to 105 °C at 10 °C/min under a nitrogen flow rate of 50 mL/min. The glass-transition temperature was extracted from the heat-flow curves.

### 5.2. Tensile Testing

Uniaxial tensile tests were conducted on pure PLA and 10 wt% CF/PLA using an LD26-series microcomputer-controlled electronic universal testing machine (Lishi (Shanghai) Scientific Instrument Co., Ltd., Shanghai, China). Dog-bone specimens were printed according to ASTM D638-22 [35], with an initial gauge length of 50 mm, grip separation of 115 mm, parallel-section thickness of 4 mm and width of 10 mm. Specimens were aligned with the loading direction to minimize bending from eccentric loading. Tests were conducted under displacement control at a crosshead speed of 2 mm/min. A contact extensometer was mounted on the gauge section to record axial strain. Load and displacement were acquired synchronously and used to calculate engineering stress-strain curves, elastic modulus, tensile strength and elongation at break. Three specimens were tested for each material, and invalid tests caused by grip slip or fracture outside the gauge section were excluded.

### 5.3. Three-Point Bending Testing

Three-point bending tests were performed on pure PLA and 10 wt% CF/PLA using the same universal testing machine. The tests followed ASTM D790-25 [36] for rectangular plastic and composite specimens. The nominal specimen dimensions were 10 mm × 80 mm × 4 mm. Actual width and thickness were measured before testing, and the support span was set according to the standard. The specimens were placed horizontally on two parallel supports, and the loading nose was aligned with the span centre. The tests were displacement-controlled at 1 mm/min with a maximum displacement of 30 mm, unless fracture or a significant load drop occurred earlier. Load-displacement data were recorded to obtain flexural stress-strain curves and calculate flexural strength, flexural stiffness and maximum deflection. During the testing process, three samples were tested for each material.

### 5.4. Quasi-Static Compression Testing

The arrow, re-entrant hexagonal, star-shaped and chiral-rotating metamaterials were compressed using a microcomputer-controlled electronic universal testing machine. Specimen dimensions and mass were measured before testing, with three samples evaluated per topology. Each specimen was positioned between parallel rigid platens with its geometric centre aligned to the loading axis. Compression was applied under displacement control at 2 mm/min to a maximum displacement of 60 mm or until full densification or load-capacity limits were reached. Load-displacement data were recorded continuously, while a camera captured cell rotation, wall bending, local buckling and progressive collapse. Engineering stress and strain were calculated from the load divided by the initial bearing area and the displacement divided by the initial height, respectively. To quantify the auxetic response, the transverse and axial engineering strains, *ε*_x_ and *ε*_y_, were obtained from the recorded compression images. The effective engineering Poisson’s ratio was calculated as *ν*_eff_ = −*ε*_x_/*ε*_y_, and the minimum value within the axial compressive strain range of 0.20 to 0.30 was extracted for each topology. Initial effective modulus, peak stress, plateau stress and densification strain were extracted from the stress-strain response, and the absorbed energy per unit volume and specific energy absorption were obtained by integrating the compression curves.

## 6. Results and Discussion

### 6.1. Thermal and Mechanical Properties of 3D Printing Materials

**Figure 4:**
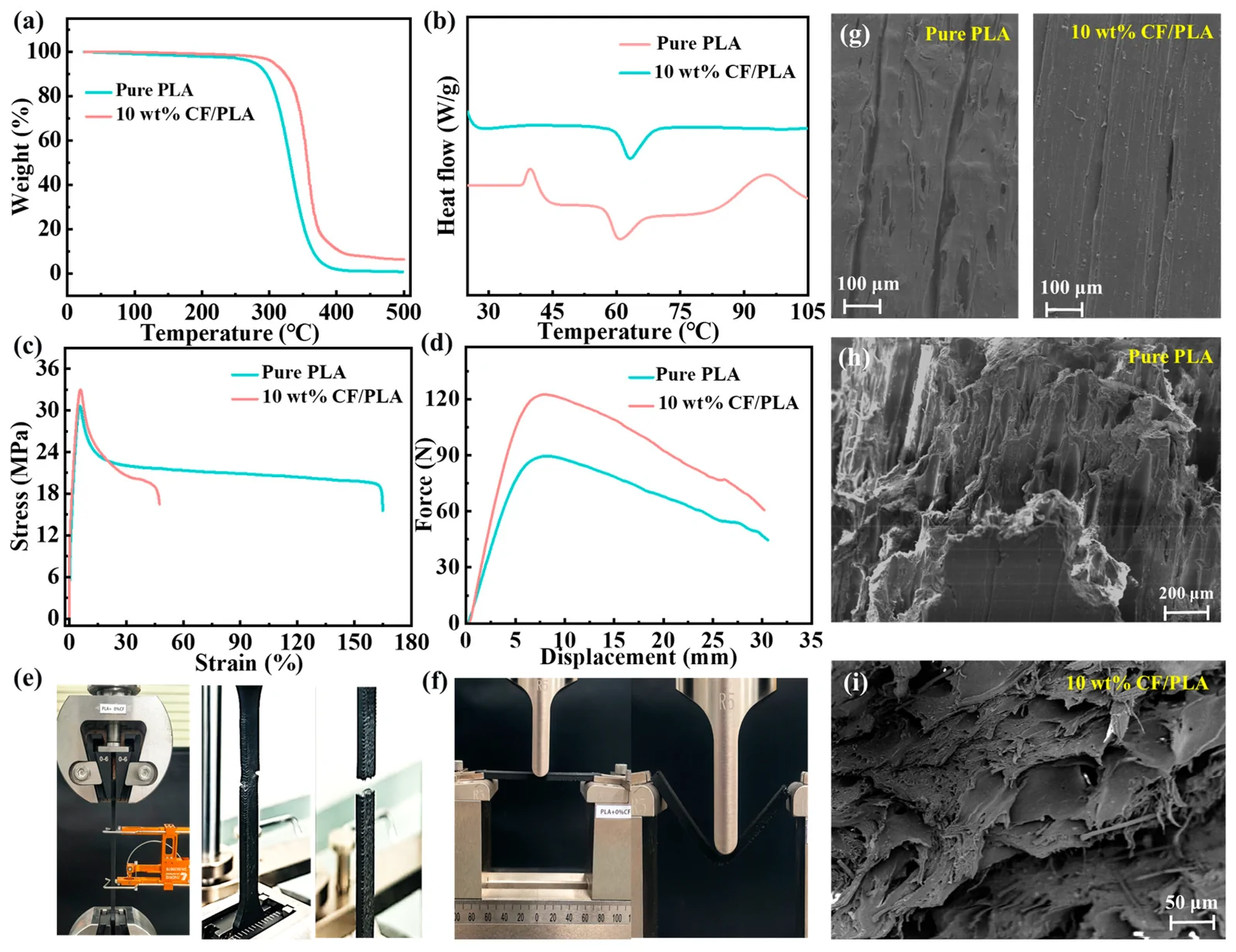
Thermal and mechanical characterization of 3D-printed PLA and 10 wt% CF/PLA: (**a**) TGA curves; (**b**) DSC curves; (**c**) tensile stress–strain curves; (**d**) three-point-bending curves; (**e**) tensile test; (**f**) three-point-bending test; (**g**) SEM images of the surface morphology of unloaded specimens; (**h**,**i**) tensile fracture morphology of pure PLA and 10 wt% CF/PLA printed specimen.

Figure 4a shows the TGA response of pure PLA and 10 wt% CF/PLA. Both materials exhibited one main degradation stage, with the principal mass loss occurring between approximately 300 °C and 400 °C. The temperature at 50% mass loss increased from about 335 °C for pure PLA to about 360 °C for CF/PLA. The residual mass at 500 °C was close to zero for pure PLA, whereas a higher residual mass remained for CF/PLA. These observations indicate that short carbon fibers delayed matrix degradation, most likely by restricting PLA chain mobility and impeding the escape of thermal-decomposition products.

The DSC curves in Figure 4b show that the glass-transition temperature increased slightly from about 60 °C for pure PLA to about 63 °C for 10 wt% CF/PLA. The upward shift suggests that the carbon fibers restrict segmental motion in the PLA matrix while strengthening the local fiber–matrix interaction. A distinct difference is also evident in the representative tensile response shown in Figure 4c. Pure PLA continued to deform over a broad plastic region after yielding, whereas the CF/PLA composite sustained a higher stress but lost its load-carrying capacity soon after the maximum stress was reached. The tensile modulus increased from approximately 2325 MPa to 3717 MPa, and the tensile strength increased from approximately 30.6 MPa to 33.0 MPa. These values correspond to increases of about 60% and 8%, respectively. The strain at break decreased from about 165% to 48%, confirming that short-fiber reinforcement increased stiffness and load transfer but reduced ductility. The measured tensile strength of the printed PLA (30.6 MPa) was lower than the supplier’s nominal value (50 MPa) primarily due to FDM-induced inter-bead voids, weaker interlayer bonding, and anisotropic thermal history during printing.

Figure 4d presents the representative three-point-bending response. Both materials showed an initial nearly linear loading stage followed by gradual softening after the peak load, while the CF/PLA specimens carried higher loads throughout the test. The peak load increased from about 90 N for pure PLA to about 123 N for CF/PLA. The material properties summarized in Table 4 indicate higher flexural stiffness and strength after carbon-fiber addition. Overall, the thermal and mechanical characterization confirms that 10 wt% short carbon fibers provided a stiffer and stronger printable matrix for auxetic metamaterials, while also making the matrix less tolerant of large tensile strains.

SEM observations in Figure 4g–i clarify the microstructural origins of the mechanical behavior. The as-printed 10 wt% CF/PLA exhibits a pronounced deposition surface texture with inter-bead voids. Upon tensile fracture, pure PLA displays a continuous morphology reflecting ductile deformation, whereas CF/PLA exhibits a rough fracture surface with micro-voids, interfacial debonding, and fiber pull-out traces. These microstructural features confirm the heightened stress concentration around fiber–matrix interfaces, providing microstructural support for the observed reduction in ductility and localized cracking.

### 6.2. Compression Behavior of Arrow and Re-Entrant Hexagonal Metamaterials

**Figure 5:**
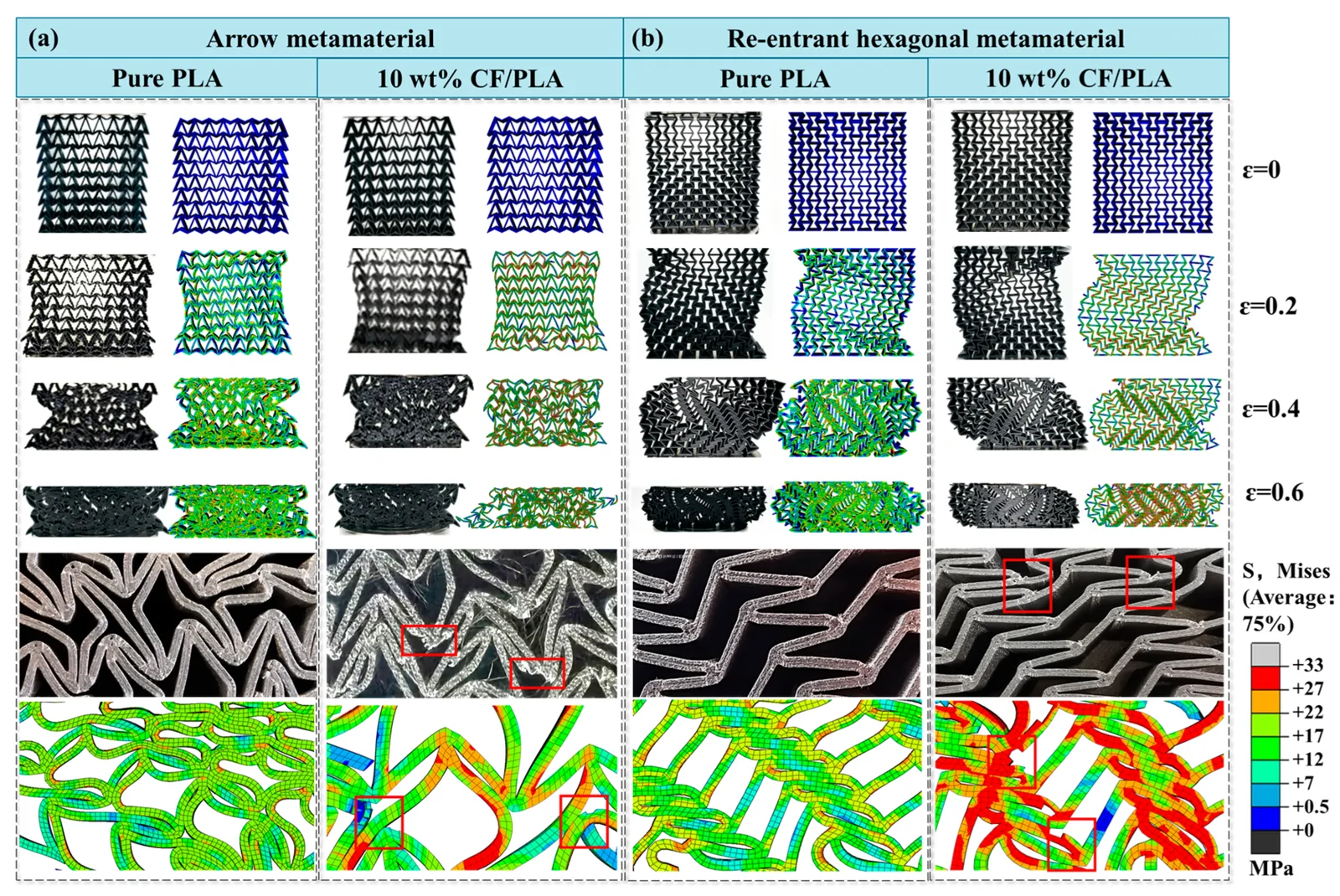
Deformation morphology and damage evolution during compression: (**a**,**b**) pure PLA and 10 wt% CF/PLA arrow and re-entrant hexagonal auxetic metamaterials.

Figure 5a compares the experimental and simulated deformation and damage evolution of the arrow metamaterials. At a compressive strain of about 0.2, the response was dominated by elastic bending of inclined cell walls and rotation at the nodes, while the global architecture remained intact. With increasing strain, the inclined walls rotated inward and underwent local buckling, producing pronounced lateral contraction. During the plateau stage, local buckling developed into coordinated folding over multiple cell rows. As compression progressed, adjacent cell walls touched and the lattice gradually compacted. Pure PLA accommodated this stage through smooth wall bending and sustained plastic folding, producing a relatively homogeneous deformation field without pronounced cracking. The CF/PLA specimen behaved differently: stress became concentrated near corners, nodal connections, and wall-contact zones, promoting crack growth, local wall rupture, and surface spalling (highlighted by the red box in Figure 5). The numerical model captured these deformation and failure features, including the overall collapse pattern, final compacted shape, and principal damage regions.

Figure 5b presents the compressive behavior of the re-entrant-hexagonal lattices. At a strain of about 0.2, the oblique ribs deflected toward the specimen center as the re-entrant joints rotated, resulting in an evident transverse contraction. A diagonal collapse zone emerged at this early stage and was observed in both the test images and numerical predictions. With further loading to a strain of 0.4, the inclined band extended through neighboring rows and triggered a stepwise loss of stability. Unlike the arrow-shaped lattice, which collapsed through relatively uniform layer-by-layer folding, the re-entrant hexagon showed a strongly localized, shear-controlled deformation mode. By a strain of 0.6, extensive rib rotation, mutual contact, interlocking, and compaction dominated the response. The pure PLA specimens accommodated these changes mainly through large plastic bending and overall shear distortion, leading to a comparatively dispersed stress distribution. The CF/PLA lattices exhibited greater load resistance, although stresses became concentrated at re-entrant corners, rib intersections, and contact points; the simulated local Mises stress reached nearly 33 MPa. Cracking first appeared around the nodal regions and subsequently advanced along the ribs, producing local wall breakage and material detachment.

### 6.3. Compression Behavior of Star-Shaped and Chiral Rotating Metamaterials

**Figure 6:**
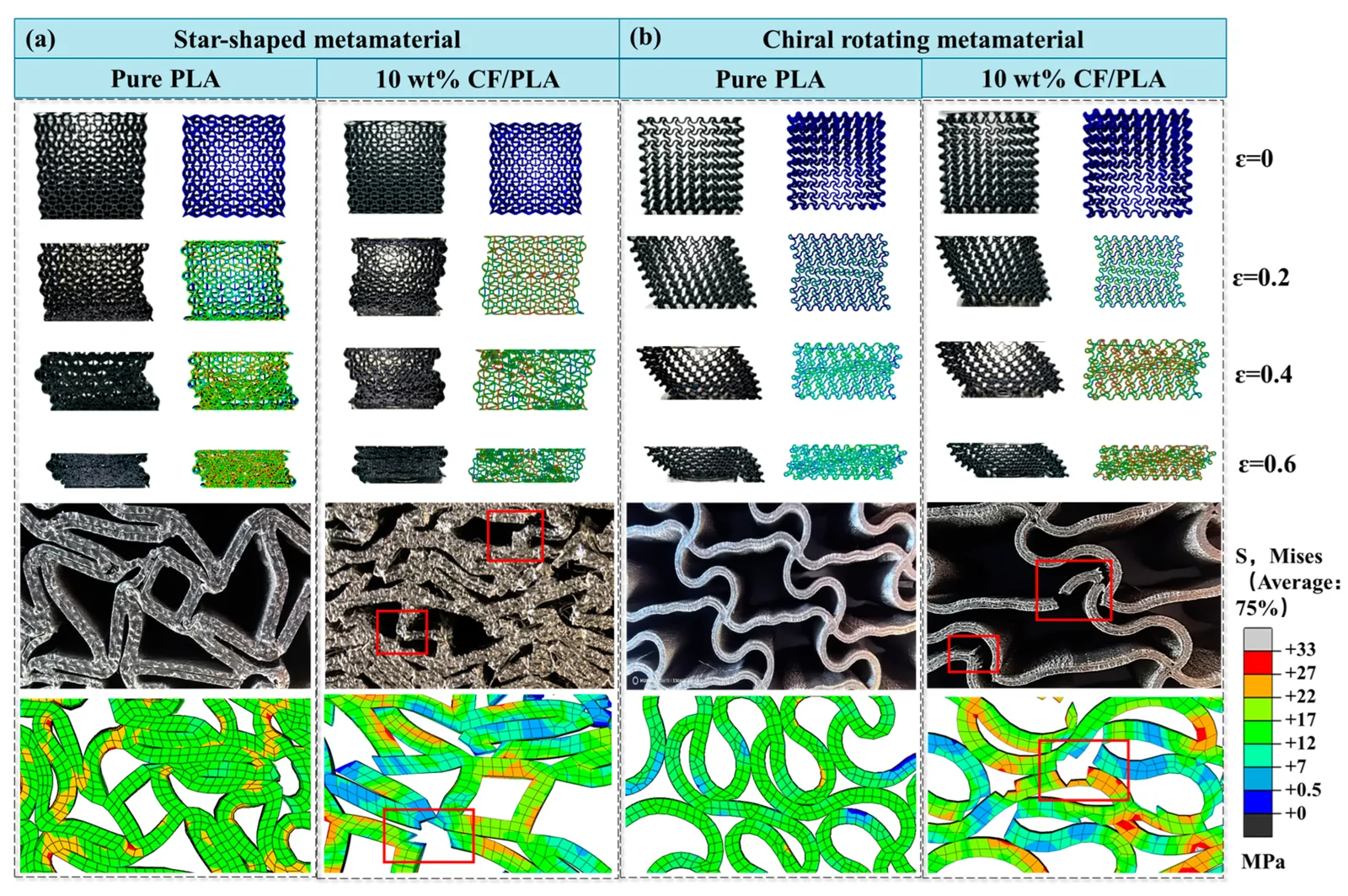
Deformation morphology and damage evolution during compression: (**a**,**b**) pure PLA and 10 wt% CF/PLA star-shaped and chiral rotating auxetic metamaterials.

Figure 6a illustrates the compression-induced deformation of the star-shaped lattices. During the initial stage, the arms rotated and bent toward the specimen center, causing a pronounced transverse contraction in both material systems. The calculated deformation pattern closely matched the experimental observations. Continued compression promoted contact between adjacent arms, after which the structure gradually shifted from nodal rotation to interlocking and compaction. Pure PLA accommodated this process through extensive plastic bending, and the highest stresses remained mainly around the arm hinges without producing visible large-scale cracking. By comparison, the CF/PLA lattice resisted a higher compressive load but showed stronger stress localization near the re-entrant corners and nodal junctions. These regions subsequently became the preferred sites for crack initiation because the reinforced material had a lower capacity for local tensile deformation (indicated by the red box in Figure 6). The numerical results reproduced the observed evolution from broadly distributed flexural deformation to concentrated damage.

Figure 6b depicts the compressive response of the chiral lattices. At low strain, deformation was governed by the coupled rotation of the central rings and flexure of the connecting ligaments. Adjacent cells rotated in opposite directions, generating a relatively uniform inward contraction across the specimen. This rotational pattern was reproduced well by the numerical model. As loading proceeded, ligament bending intensified, neighboring cell walls came into contact, and the lattice progressively approached a compacted state. The pure PLA specimens sustained this deformation through flexible ligament bending and large nodal rotation, resulting in a broadly distributed stress field. In the CF/PLA lattices, the stiffer ligaments transferred load more effectively but also produced markedly higher local Mises stresses during rotation and contact. Interfacial stress concentration and geometric constraints nevertheless promoted cracks at ligament-node connections.

The minimum effective Poisson’s ratios are summarized in Table 5. Negative values were obtained for all four topologies in both material systems, quantitatively confirming their auxetic behavior. The minimum effective Poisson’s ratios became less negative for all four topologies after short-carbon-fiber reinforcement, indicating a reduced magnitude of the auxetic response within this strain range.

### 6.4. Energy Absorption Performance

**Figure 7:**
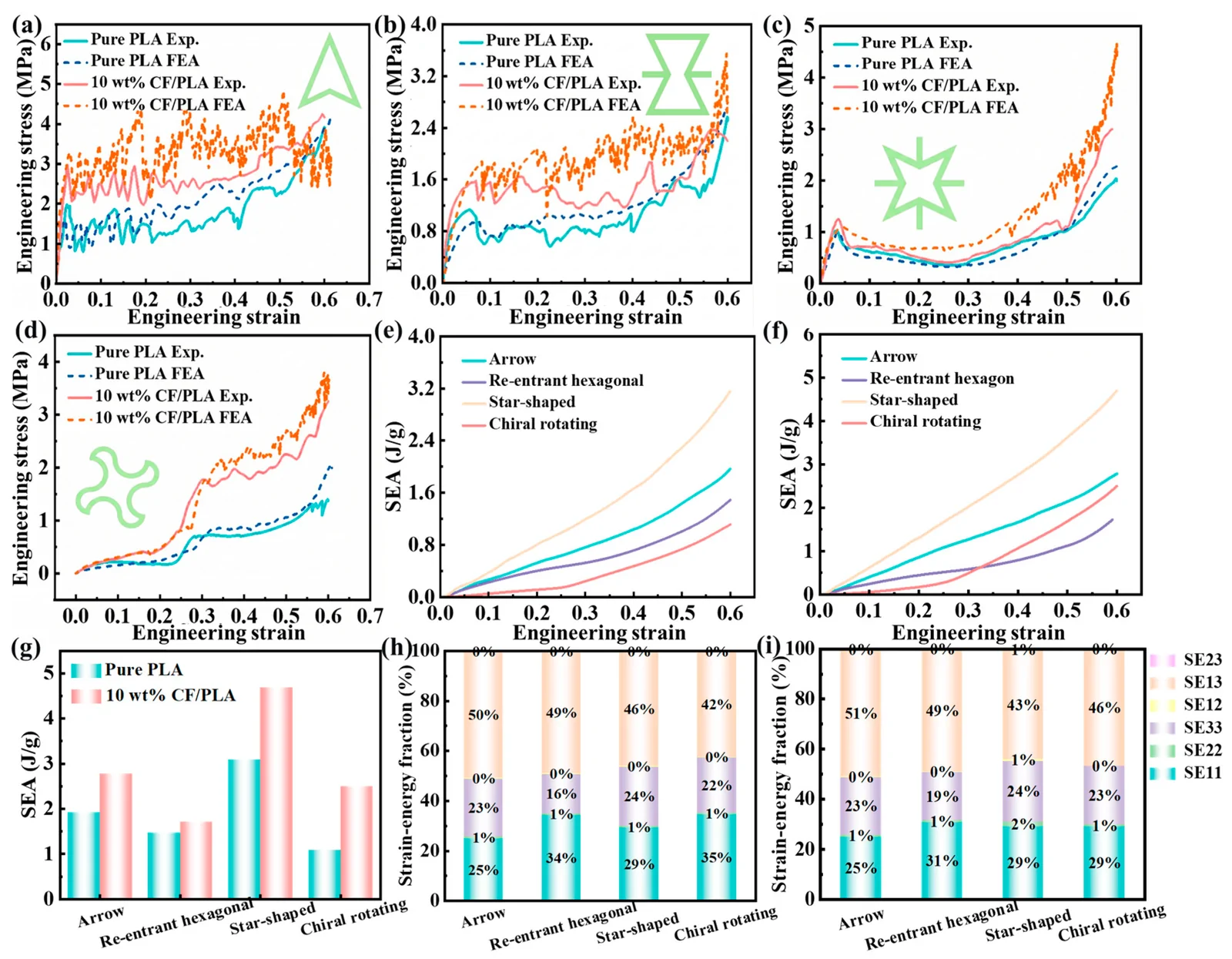
Energy-absorption comparison of the four auxetic metamaterials for PLA and 10 wt% CF/PLA: (**a**–**d**) compression stress-strain curves for arrow, re-entrant hexagonal, star-shaped, and chiral rotating metamaterials; (**e**,**f**) specific energy absorption versus engineering strain for pure PLA and 10 wt% CF/PLA, respectively; (**g**) specific energy absorption at an engineering compressive strain of 0.60; (**h**,**i**) strain-energy component fractions for PLA and 10 wt% CF/PLA.

Figure 7a–d compare the experimental and simulated stress-strain curves of the four auxetic topologies. Although each lattice exhibits the characteristic sequence of elastic loading, progressive collapse, and densification, the shape and stability of the plateau region are strongly architecture-dependent. The arrow lattice develops an early load peak: approximately 2.0 MPa for pure PLA and 2.8 MPa for 10 wt% CF/PLA. The subsequent plateau lies near 1.0–1.8 MPa for PLA and 2.2–2.8 MPa for the reinforced material. This response reflects bending of the inclined ribs, rotation at the re-entrant joints, and successive cell folding, while extensive wall contact produces pronounced hardening beyond a strain of roughly 0.45. For the re-entrant hexagonal lattice, pure PLA reaches an initial maximum of about 1.5 MPa before settling into a lower plateau associated with rib rotation and the growth of a diagonal collapse band. Carbon-fiber reinforcement raises the overall load level, with stress exceeding 4 MPa near full compaction, although intermittent buckling and localized damage lead to larger oscillations. The star-shaped lattice maintains a relatively long, low-load plateau because arm rotation and central-junction bending accommodate substantial deformation before rapid compaction begins at strains of about 0.45–0.50. In contrast, the chiral lattice shows the most gradual initial rise, as its curved ligaments deform mainly through rotation and flexure. Its resistance increases markedly at intermediate and large strains after CF addition, owing to the higher ligament stiffness and more effective force transmission. Overall, the finite-element predictions reproduce the principal peaks, collapse fluctuations, and onset of densification for all four designs. The agreement is better for pure PLA, whereas local maxima in CF/PLA are commonly overpredicted. The SEM observations reveal microstructural heterogeneity in the actual CF/PLA material, including local pores, heterogeneous fiber distribution, and inter-bead gaps. In contrast, the finite-element model assumes an idealized homogeneous material condition. This difference may explain the overprediction of the CF/PLA response in the simulations.

Figure 7e,f show the evolution of specific energy absorption with compressive strain for pure PLA and 10 wt% CF/PLA metamaterials. To eliminate mass discrepancies from tessellation and relative density, absorbed energy was normalized by specimen mass. For pure PLA, the star-shaped topology exhibited the highest SEA (3.16 J/g at *ε* = 0.6), followed by the arrow (1.96 J/g), re-entrant hexagonal (1.48 J/g), and chiral rotating (1.08 J/g) designs. Incorporating 10 wt% carbon fibers accelerated SEA accumulation across all geometries. The star-shaped CF/PLA structure delivered the highest overall SEA of 4.70 J/g (+48.7%) via coordinated node-locking. Notably, the chiral rotating topology achieved the largest relative improvement of 132% (2.51 J/g), surpassing the re-entrant hexagonal lattice (1.71 J/g) beyond *ε* = 0.32.

The simulated strain-energy components in Figure 7h,i clarify the dominant strain-energy distribution. For the arrow topology, shear strain energy SE13 was the largest component in both pure PLA and CF/PLA, contributing about 50% and 51%, respectively. It was followed by in-plane normal strain energy SE11 and through-thickness/vertical strain energy SE33. Across the four architectures, SE13 remained the principal energy-storage and dissipation channel, with contributions of about 42–51%. The star-shaped and chiral structures showed larger SE33 bending contributions, consistent with their node-bending and ligament-rotation mechanisms. The similarity of the energy-component distributions before and after fiber addition indicates that topology governed the partition of deformation energy, whereas carbon-fiber reinforcement primarily changed the stress level, stiffness, and damage localization.

## 7. Conclusions

This study combined experiments and finite-element simulations to compare four auxetic topologies (arrow, re-entrant hexagonal, star-shaped and chiral rotating) printed with pure PLA and 10 wt% CF/PLA. Carbon-fiber addition improved the matrix properties, increasing the 50% mass-loss temperature from about 335 °C to 360 °C and the tensile modulus from approximately 2325 MPa to 3717 MPa. The reinforced lattices also showed higher plateau stresses and greater energy absorption. Their crushing behavior, however, depended strongly on cell architecture. The arrow design collapsed through rib rotation and progressive folding, whereas the re-entrant hexagonal lattice developed a diagonal shear band. In the star-shaped structure, deformation was distributed among multiple joints before rapid compaction, while the chiral design accommodated loading mainly through bending and rotation of curved ligaments. Although CF reinforcement enhanced stiffness and resistance to collapse, it reduced the ability of the material to sustain large local deformation, resulting in crack concentration around hinges and re-entrant corners. Among the tested designs, the star-shaped CF/PLA metamaterial delivered the best overall energy absorption (SEA~4.7 J/g), while the chiral rotating metamaterial showed the largest relative improvement after reinforcement (SEA increase > 130%). The numerical model captured the main collapse and damage patterns and indicated that shear strain energy contributes 42–51% of the total stored deformation energy. These results reveal a trade-off between material strengthening and topology-induced damage localization. This material-structure interplay provides a practical basis for lightweight crash-mitigation and impact-protection components in aerospace and related fields.

## Figures and Tables

**Table 1:** Geometric parameters, array configurations, cell dimensions, and specimen masses of the printed metamaterials.

Topology Type	Cell Parameters	Array Grid	Cell Dimensions	Specimen Mass (Pure PLA)	Specimen Mass (10 wt% CF/PLA)
Arrow	*a*_1_ = 13.5 mm; *θ*_1_ = 46°; *α*_1_ = 65°; *t*_1_ = 1 mm; *W* = 20 mm	8 × 8	13.5 mm × 16 mm × 20 mm	62.82 g	64.47 g
Re-entrant hexagonal	*a*_2_ = 14.5 mm; *b*_2_ = 13.5 mm; *θ*_2_ = 60°; *t*_2_ = 1 mm; *W* = 20 mm	7 × 8	14.5 mm × 13.5 mm × 20 mm	62.52 g	63.71 g
Star-shaped	*a*_3_ = 15 mm; *θ*_3_ = 40°; *t*_3_ = 1 mm; *W* = 20 mm	7 × 7	15 mm × 15 mm × 20 mm	68.28 g	69.86 g
Chiral rotating	*a*_4_ = 17.7 mm; *R*_4,int_ = 2.9 mm; *R*_4,ext_ = 2.9 mm; *t*_4_ = 1 mm; *W* = 20 mm	8 × 8	17.7 mm × 17.7 mm × 20 mm	61.37 g	62.92 g

**Table 2:** Mesh statistics of the four auxetic metamaterial models.

Topology Type	Characteristic Element Size (mm)	Total Number of Elements	Total Number of Nodes
Arrow	0.50	475,440	698,353
Re-entrant hexagonal	0.50	466,880	683,716
Star-shaped	0.50	601,080	862,927
Chiral rotating	0.50	505,800	725,126

**Table 3:** Material parameters used in the finite-element simulations.

Parameter	PLA	10 wt% CF/PLA
Density (kg/m^3^)	1260	1280
Elastic modulus (GPa)	2.36	3.75
Poisson’s ratio	0.33	0.31
Plasticity model	Linear Drucker-Prager	Linear Drucker-Prager
Friction angle	30°	20°
Flow-stress ratio	0.9	1.0
Dilation angle	5°	10°
Damage model	Ductile damage	Ductile damage
Fracture energy, Gf (J/m^2^)	300	270

**Table 4:** Material properties of pure PLA and 10 wt% CF/PLA.

Material	Tensile Modulus (MPa)	Tensile Strength (MPa)	Elongation at Break (%)	Flexural Modulus (MPa)	Flexural Strength (MPa)
Pure PLA	2325 ± 21	30.6 ± 1.2	165 ± 5	1725 ± 11	53.4 ± 2.6
10 wt% CF/PLA	3717 ± 31	33.0 ± 2.1	48 ± 2	2390 ± 16	73.2 ± 3.8

**Table 5:** Minimum effective Poisson’s ratios of the four auxetic topologies.

Topology Type	Pure PLA	10 wt% CF/PLA
Arrow	−0.25 ± 0.03	−0.20 ± 0.02
Re-entrant hexagonal	−0.32 ± 0.02	−0.25 ± 0.03
Star-shaped	−0.20 ± 0.03	−0.13 ± 0.04
Chiral rotating	−0.38 ± 0.02	−0.23 ± 0.02

## Data Availability

The original contributions presented in this study are included in the article. Further inquiries can be directed to the corresponding authors.
